# A Review of the Phytochemistry and Antimicrobial Properties of *Origanum vulgare* L. and Subspecies

**DOI:** 10.22037/ijpr.2020.113874.14539

**Published:** 2021

**Authors:** Saba Soltani, Abolfazl Shakeri, Mehrdad Iranshahi, Motahareh Boozari

**Affiliations:** a *Department of Food and Drug, Control Laboratory of Food and Drug, Mashhad University of Medical Sciences, Mashhad, Iran. *; b *Department of Pharmacognosy, School of Pharmacy, Mashhad University of Medical Sciences, Mashhad, Iran. *; c *Biotechnology Research Center, Pharmaceutical Technology Institute, Mashhad University of Medical Sciences, Mashhad, Iran.*

**Keywords:** Origanum vulgare L., Phytochemistry, Traditional uses, Antimicrobial activities

## Abstract

*Origanumvulgare *L. (*O. vulgare*) is an important medicinal herb of the family Lamiaceae. In the current study, we explained the critical evaluation of traditional uses, the phytochemistry and the antimicrobial properties of *O. vulgare* and its subspecies, with a focus on the mechanisms of actions of the most important phytochemicals from *O. vulgare* subspecies. The most important phytochemicals of *O. vulgare* are volatile (essential oil) and non-volatile phenolic compounds (phenolic acids & flavonoids). The constituents of the *O. vulgare* essential oil (EO) include high percentages of thymol and carvacrol with excellent antimicrobial activity alone or in combination with other antibiotics. Interesting results have been reported the remarkable antimicrobial activities of infusion or tea products of *O. vulgare* with a high amount of EO against multidrug-resistant bacterial and fungal microorganism (such as *Escherichia coli, Staphylococcus aureus, Candida albicans* and *Pseudomonas aeruginosa*). The most important antibacterial mechanisms of *O. vulgare* are enzyme inhibition, efflux pump inhibition, ATP depletion, biofilm formation inhibition and cytoplasmic membrane damage. The antimicrobial activity of the *hirtum* subspecies has been confirmed in different *in-vitro *and *in-vivo *studies. The present review confirms the clinical and preclinical research showing the *O. vulgare* and its subspecies antimicrobial effects.

## Introduction

Members of the genus *Origanum* comprise the most important herbaceous and aromatic medicinal plants from the family Lamiaceae that distributes in warm and mountainous areas. *O. vulgare* L. (known as “oregano”) as the most diverse species in the genus are spread in the Mediterranean region and Western and Southwestern Eurasia region (1). Ietswaart identified morphologically six subspecies of *O. vulgare *(1):* glandulosum *(Desf.) Ietsw.,* gracile *(K.Koch) Ietsw., *hirtum *(Link) Ietsw., *virens *(Hoffmanns. & Link) Ietsw., *viridulum* (Martrin-Donos) Nyman., and *vulgare*. These subspecies are well accepted in 2013 with “The Plant List” (www.theplantlist.org). In Iran *O. vulgare* includes three subspecies (subsp. *viride*, subsp. *vulgare* and subsp. *gracile*) that grow mainly in northern parts of the country (2) and their morphological diversity of wild varieties reviewed by Andy *et al. *(3). The term “oregano” can be confusing because it is known to be a vernacular term for many other species, for example, Mexican oregano (*Lippia graveolens* Kunth). Therefore, for more clarity we stick to the term *Origanum vulgare* L. To date, over 100 volatile and non-volatile ingredients have been recognized in the oil and various extracts of *O. vulgare*. Based on hydrophilic and hydrophobic features, there are exist two main groups of phytochemicals in *O. vulgare*, include essential oils (EOs) and phenolic compounds (flavonoids and phenolic acids). Others biological active compounds consist of terpenoids, tannins and sterols (4). Different subspecies of *O. vulgare *are found in wild varieties on various soils with different fertility and rather low temperatures, but many other ones can be cultivated as medicinal, culinary and garden plants and play a very important role in the economy and constitutes one of the most cultivated aromatic plants worldwide. One of the largest global markets is related to *O. vulgare* ssp. *hirtum* (known as Greek oregano) due to its perfect quality and high EO concentration which is predominantly expanding in Turkey, Greek, Cyprus and Italy (5, 6). Some of the uses for *O. vulgare* in traditional medicine are respiratory disorders, stomachache, painful menstruation, rheumatoid arthritis, analgesics, nutritive disturbance and urinary problems as a diuretic and antiurolithic (7, 8). From the last two decades, following the increasing of antibacterial resistance as a menace to global health, the interest of scientists has been devoted to antimicrobial studies (9). According to the literature, about two-thirds of clinically antibacterial therapies are designed on the basis of natural products (10). Different studies show that essential oils are safe antibacterial compounds in combating infections (11). Eos constituents that can inhibit the growth of bacteria, yeasts and moulds and resistance to them could be more difficult than to single antibiotic molecule (12). Various species of *O. vulgare* are among the most studied plants due to the potential antibacterial effects that are different based on the species of microorganisms (wild, reference, drug-sensitive, or resistant) and the type of plant extraction (EOs or various extracts), and it should be taken in this regard whenever exploring the plants’ potential for developing new antimicrobial drugs. Regarding the importance of this species, the biological effects of EOs, extracts or the main constituents have been previously reviewed (13). However, this review article concentrated on the variation of the non-volatile and volatile ingredients of *O. vulgare, *their traditional therapeutic effects and focusing on antimicrobial activities. 

Search method 

The current review consists of scientific studies regarding the *O. vulgare* subspecies published between 2000 and 2020. At the first of this study, 307 papers were evaluated and among these studies, 111 references focusing on ethnopharmacology data, phytochemistry and pharmacology studies of the *O. vulgare* and its subspecies were selected. Another 56 papers were used to complete the current review article. Six subspecies were indicated according to the plant list website classification (www.theplantlist.org). Furthermore, an older text from 1990 about the traditional uses of *O. vulgare* has also been mentioned and studied. Information was gathered by searching the internet (PubMed, Francis & Taylor, Wiley, Scopus, Web of Science, ACS, ScienceDirect, Springer, Google Scholar and The Plant List Database). The authors have also checked the libraries, Iranian traditional books and some thesis works that were considered firstly. The data from patents, congress abstracts and symposiums were omitted because of the uncompleted source in comparison to data from full papers and books. All related databases were searched for the terms “*Origanum vulgar*e” and its subspecies, ” antimicrobial”/”phytochemistry”/”traditional”. 

Traditional uses

For centuries, *O. vulgare* has traditionally been used to flavor foods and treatment of various diseases due to the high percentage of their EO (14). In the 7^th^ century B.C, *O. vulgare *was used to flavor fish, meat, vegetables and wine (15). The useful subspecies of *O. vulgare *in culinary include ssp. *gracile*, ssp. *glandulosum, *ssp. *hirtum* (16). Some of the uses for *O. vulgare* in traditional medicine are respiratory disorders, stomachache, painful menstruation, rheumatoid arthritis, nutritive disturbance and urinary problems as a diuretic and antiurolithic. Aerial parts of the plant were mostly used. In 2018, Bahmani *et al. *reviewed the therapeutic effects of *O. vulgare* based on Iran’s ethnopharmacological documents (17). All of them reported that in Iran, *O. vulgare* is used for flavoring in cooking and in traditional medicine as a tonic, expectorant, carminative, stimulant and antibacterial agent (18, 19).

The forms of consumption are very diverse according to the symptoms, including tea or tincture that is used against cold and digestive or respiratory disorders and improve the general health of the body (7). Decoction or infusion preparation of *O. vulgare* has been used for expectorant, antiseptic, digestive aid and antispasmodic properties (20). Pieroni *et al. *reported on smoke inhalation to relieve toothache (21). The routinely used *O. vulgare* subspecies, their consumed part, methods of preparations and important traditional features are summarized in Table S1 (in supplementary file).

Phytochemistry

*Volatile compounds*

**Figure 1:**
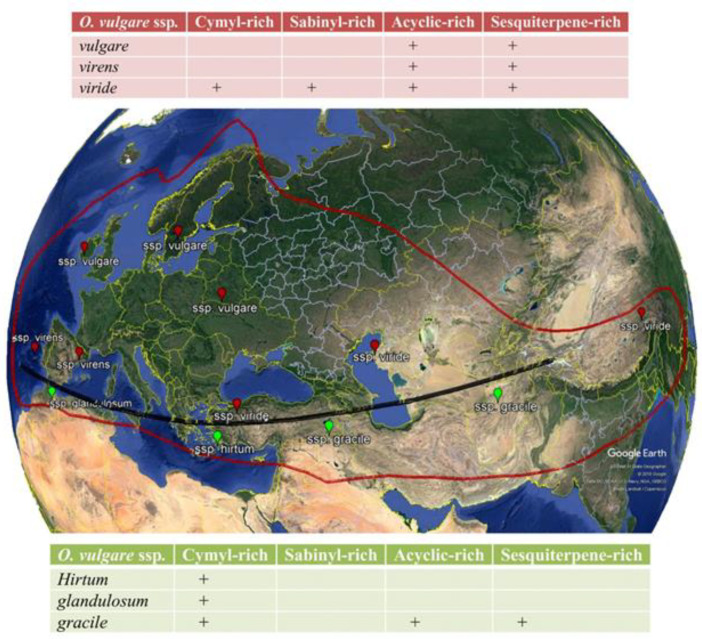
Simplified presentation of the distribution of the six *Origanum vulgare* ssp. Above the black line, the taxa are poor in essential oil, whereas the essential oil rich subspecies of *O. vulgare* occur below the line (reflecting data collected from Kokkini, 1996 and Ietswaart, 1980)

**Figure 2:**
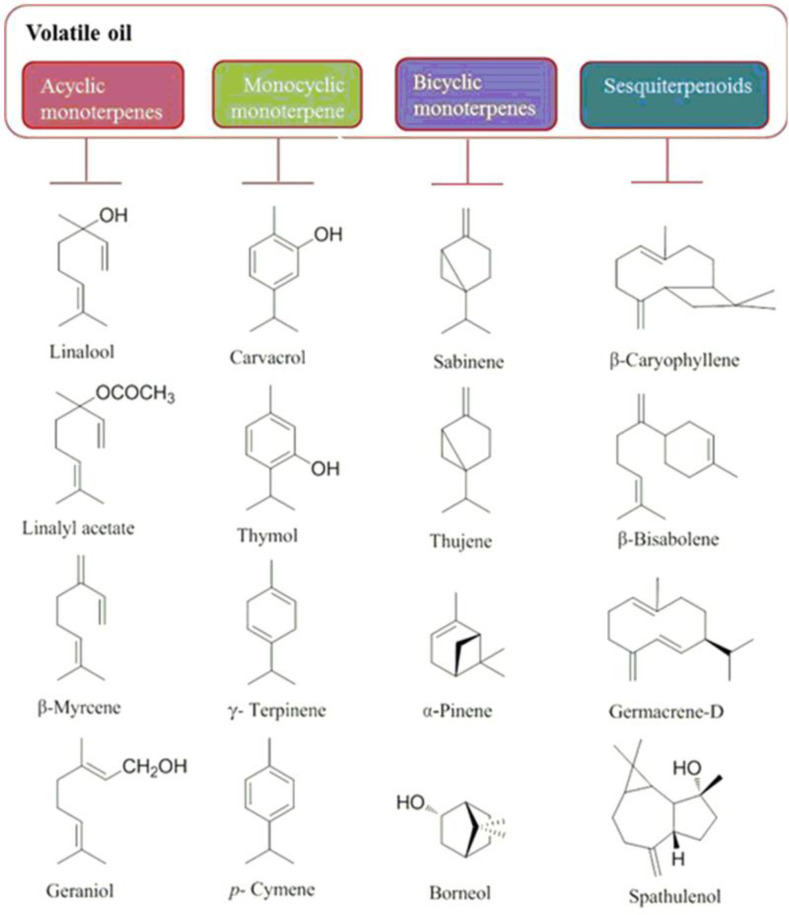
Chemical structures of main volatile compounds of *O. vulgare*

Essential oils are the main group among many compounds obtained from *O. vulgare*. As demonstrated in Table S2 (in supplementary file) and Figure 1, regarding the geographic origin, extraction method, plant’s developmental stage, growing conditions and harvest time, oil yield and volatile compositions is diverse. Therefore, a detailed comparison between various reports is very difficult. In general, *O. vulgare* EO is a great source of monocyclic monoterpenes (thymol, *γ*-terpinene, carvacrol, and *p*-cymene), acyclic monoterpenes (geraniol, linalyl acetate, linalool and *β*-myrcene) and bicyclic monoterpenes (sabinyl compounds) and sesquiterpenoids (*β*-bisabolene, *β*-caryophyllene, spathulenol and germacrene-D) have also been reported depending on the chemotype (Figure 2). Several studies have reported that subspecies grown in northern Mediterranean areas are poor sources of volatiles (with complex compositions of phenolic monoterpenoids, acyclic compounds, camphane type compounds, sabinyl-compounds and larger numbers of sesquiterpenes); whereas, those grown in southern regions are enrichment in EO with phenolic monoterpenoids (cymyl compounds), mainly carvacrol or thymol that can constitute up to 70% of the total oil (Figure 1) (22-26). Furthermore, *γ*-terpinene and *p*-cymene have been reported in considerable amounts with different concentrations, attributed to the reverse relationship with carvacrol (γ-terpinene converts to p-cymene autoxidation and subsequently converts to carvacrol by hydroxylation) (27). The main ingredients of ssp*. glandulosum* EO is thymol, carvacrol and their methyl ethers (28). The EO of the same subspecies from Tunisia (29) the percentage of carvacrol was high, while from another region of the same country (30) and also in Algeria (31), the percentage of thymol and* p*-cymene was higher than other EO ingredients. The main components of the ssp*. gracile* EO collected from Iran were carvacrol (60.6%), *γ*-terpinene (16.64%) and *p*-cymene (13.54%) (4, 32); and EO of this subspecies from Turkey consists of *β*-caryophyllene (17.54%) and germacrene D (12.75%) (33), whereas EO of ssp*. gracile* from France identified by high percentage of the sabinene (26.0%), germacrene D (13.7%) and *β*-caryophyllene (6.6%) (34). According to the literature, the ssp*. hirtum* has a higher EO yield than other *O. vulgare* ssp*.* In 2014, the carvacrol and thymol chemotypes were characterized (35). In these chemotypes, usually, the percentage of carvacrol is high and the percentage of thymol was low (36), while another study showed the main components of EO from Turkey is linalool (96.31%) (37). As shown in Table S2, ssp*. virens* has a high diversity in EO and the main ingredients of EO from different regions are carvacrol, linalool, thymol,* α*-bisabolene, germacrene D and *γ*-terpinene. The thymol (58%) (38) and carvacrol chemotype (68%) have been reported from Portugal (39, 40), whereas linalool chemotype (76.8%) (41, 42) have collected in Mediterranean regions and Spain. Moreover, Germacrene D chemotype (34) has been reported in France. The Iranian species of *O. vulgare *were characterized by the amount of α-bisabolene and sabinene (4) in oils. *γ*-Terpinene chemotypes were collected from Corsica (20.1%) and Central Portugal (34.2%) (40, 43). EO of ssp* viridulum* from Turkey has a high percentage of caryophyllene oxide (25.01%) and linalool (8.32%) (44). Other researches in Iran and Balkan demonstrated that thymol is the major constituents in both oils, followed by 4-terpineol and *γ*-terpinene (45, 46). In 1998, Chalchat showed that ssp. *vulgare*, incorporate at least nine chemotypes of EO, including: thymol, sabinene, *O*-cymene, *β*-caryophyllene, germacrene D, *β*-ocimene, terpinen-4-ol, spathulenol and *cis*-sabinene hydrate as shown in Table S2 (47). 

*Non-volatile phenolic compounds*

A comprehensive overview of phenolic ingredients of *O. vulgare* with different origins is summarized in Table S3 and Figures S1 and S2 (in supplementary file). The major phenolic acid **(1-12)** that has been identified in *O. vulgare* species is rosmarinic acid **(12)** (44, 48-53). Both free flavonoids (flavones, flavonols, flavanones and dihydroflavonols) and flavonoid glycosides are present in *Origanum* species (54). The most abundant flavonoids of *O. vulgare* are flavons. In addition, 6-substituted and 6, 8-disubstituted flavonoids are uncommon elsewhere, present in the genus (48, 55 and 56). A number of *O*-glycosides and *C*-glycosides have been found in *O. vulgare*. Luteolin **(36) **is the most common aglycone, followed by apigenin **(35)**; most sugar moieties are glucosides and glucuronides (54). The cultivar, geographical, environmental factors and different experimental protocols can affect the concentration and distribution of compounds in *O. vulgare*. Therefore, a detailed comparison between various reports is very difficult. For example, rosmarinic acid exhibited different contents between various chemotypes within the species of ssp. *hirtum* and European *O. vulgare *were ranging from 13.73 to 63.69 mg/g on a dry weight basis (57); these results showed a broader range of rosmarinic acid in comparison with Austrian *O. vulgare *ssp.* vulgare* chosen plants of 19 populations (9.4 to 37.2 mg/g dry mass) (24). Liang *et al. *(2010) identified a new phenolic glucoside, origanoside **(15)**, from the ethyl acetate soluble part of the methanol extract of *O. vulgare* (58). Zhang *et al. *(2014) also isolated six new phenolic compounds **(18, 19, 57-60)** along with known ones **(3, 4, 12 and 15)** from the ethanol extract (59). Rosmarinic acid methyl ester **(13)** was isolated from *O. vulgare*, which exhibited depigmentation activity (60). Two protocatechuic acid ester derivatives, origanol A **(16)** and origanol B **(17)** had been reported from the methanolic extract of O. vulgare collected from India (61). Liu and coworkers identified three new polyphenolic compounds, origanine A−C **(20-22)** (62). In 2003, a novel dihydrobenzodioxane derivative, origalignanol **(23)** and known polyphenolic compounds include salvianolic acid A **(24)**, salvianolic acid C **(25)**, lithospermic acid **(26)**, apigenin7-O-D-glucuronide **(51)**, luteolin **(36)**, luteolin 7-O-D-glucopyranoside **(45)**, luteolin7-O-D-glucuronide **(50)** were isolated from the aqueous ethanolic extract of *O. vulgare *(63). 

*Triterpenoids*

The major triterpenoids that have been reported from *O. vulgare* are pentacyclic triterpenoids such as ursolic and oleanolic acids that are common to most Labiatae, whereas diterpenoids have not been found in *O. vulgare *(54). Rao *et al. *(2011) reported the presence of ursolic acid, oleanolic acid, *β*-sitosterol and triacontanol in an ethanolic extract of *O. vulgare* from India (61). Moreover, Baranauskaite *et al. *(2016) reported the presence of ursolic acid and oleanolic acid from ssp.* hirtum* by maceration in ethanol/non-aqueous solvent (glycerol or propylene glycol) (64). Assiri *et al. *(2016) analyzed the cold-pressed oil to determine lipid profile, fatty acid, tocols and phenolic contents. The neutral lipids exhibited the maximum content, then glycolipids and phospholipids. The main fatty acids included linoleic, oleic, stearic and palmitic acids. Tocols include *γ*-tocopherol, *α*-tocotrienol and *γ*-tocotrienol with 32.1%, 25.8% and 21.3% of total measured tocols, respectively (65). The FTIR analysis of *O. vulgare* seeds demonstrates the existence of alkenes, aliphatic fluoro compounds, alcohols, ethers, carboxylic acids, esters, hydrogenated alcohols and phenols (66). Koukoulitsa *et al. *(2006) have also isolated two polar compounds (12-hydroxyjasmonic acid 12-O-β-glucopyranoside and *p*-menth-3-ene1, 2-diol 1-O-β-glucopyranoside) from the aerial parts of ssp. *hirtum* growing uncultivated in Greece (67). It was reported that hexane extract of ssp. *viridulum* contained fatty acids and hydrocarbons such as hexadecanoic acid methyl ester, 9,12-octadecadienoic acid methyl ester, 9,12,15-octadecatrienoic acid methyl ester, cyclotetracosane and 1-eicosanol (44).

**Antimicrobial activity**

**Figure 3:**
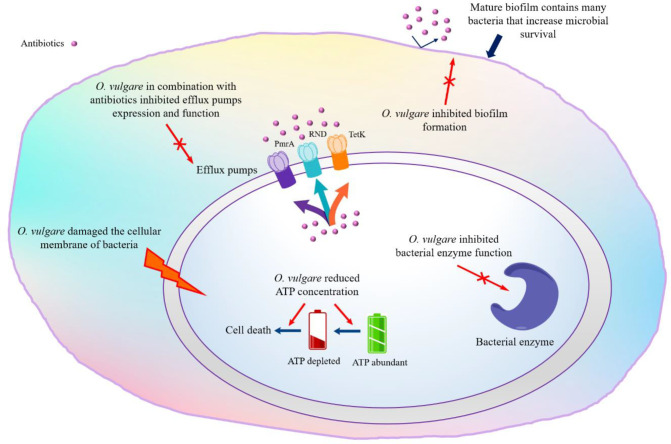
Different mechanisms of *O. vulgare* antibacterial activity

Various studies evaluated the inhibitory effects of EOs, extracts or the main constituents from *O. vulgare* against different pathogenic bacteria. Diverse mechanisms of an EO activity on bacterial cells have been proposed to explain the antibacterial activities (68). Schematic Figure 3 illustrates different mechanisms of *O. vulgare* antibacterial activity.

*Mechanisms of action*

Bacterial enzyme inhibition: One of the proposed mechanisms is inhibition the production or activity of bacterial enzymes (such as lipase and coagulase) that was mediated by EO of *O. vulgare* (at 0.03 and 0.015 μL/mL) against *S. aureus *(69). 

Efflux pumps inhibition: Potential antibacterial synergy of EOs in combination with antibiotics to inhibition of efflux pumps that is another possible mechanism of action (70), is measured by fractional inhibitory concentration index (FICI).* O. vulgare* EOs in combination with ciprofloxacin and ethidium bromide, exerted synergistic (FICI from 0.22 to 0.75) activity against fluoroquinolone resistant *Streptococcus pneumoniae* clinical isolates by inhibition of the PmrA efflux pump gene expression (71). In contrast, results by Perrin *et al. *did not show any additive or synergistic effect between EO and antibiotics against the model *strain Burkholderia cenocepacia* J2315. The obtained data showed an intracellular mechanism of action and the addition of the efflux pumps inhibitor (Phe-Arg -naphthylamide dihydrochloride, which acting on RND efflux pumps) significantly increased EO activity depend on the inactivation of different cellular, molecular targets (72). Furthermore, co-administration of tetracycline with *O. vulgare* EO (fourfold), carvacrol and thymol (twofold) exerted synergistic activity against *S. aureus* by inhibition of the TetK efflux protein (73). In addition, a significant synergistic effect between ciprofloxacin and phenolic (FICl < 0.5), nonphenolic (FICI > 4.0, antagonistic activity) fractions and volatile oil (FICl < 0.5) against *S. typhi *was reported (74). 

Antibiofilm agents: Another antibacterial mechanism of EO is biofilm eradication; for example, *O. vulgare* EO (MIC: 0.25-0.5 mg/mL) acts as a potent antibiofilm agent of *S. pyogenes* (at concertation of 0.5 mg/mL) with dual actions, preventing and eradicating. This biofilm inhibition is attributable to the killing of its planktonic cells (time to kill 99.9%, 5 min) (75). When screening 79 essential oil for antibiofilm ability against UPEC (uropathogenic Escherichia coli), Lee *et al. *(2017) found that *O. vulgare* EO, carvacrol and thymol noticeably decreased fimbriae production and swarming motility of UPEC at sub-inhibitory concentrations (<0.01%) and their results showed that the hemagglutinating ability of UPEC in the presence of carvacrol and thymol decreased and UPEC easily killed by human whole blood (76). 

Effect on the cytoplasmic membrane: Some studies considered the correlation between antimicrobial properties of EO and its phenolic compounds (carvacrol and thymol). For investigation of the antibacterial mechanism of carvacrol and thymol (with the same system of delocalized electrons) against *Bacillus cereus, *liposomal models were used. Carvacrol damaged the cellular membrane and reduced the pH gradient in the cellular membrane that leads to the proton motive force, reduction in the ATP pool and cell death (77). Khan *et al. *(2017) showed that carvacrol and thymol exhibited potent bactericidal (IC_50_: 65 and 54 µg/ml, respectively) and antibiofilm activity (at concertation of 100 μg/ml) against *Streptococcus* *mutans *(78). The same research also demonstrated the growth inhibition of *E*. *coli*, *Pseudomonas aeruginosa*, *Micrococcus* *luteus*, and *S*. *aureus* at IC_50_ values from 107-286 μg/mL for aqueous distillates (carvacrol 92.5%) and at IC_50_ values from 214-383 μg/mL for volatile oil from the aerial parts (carvacrol 70.2%), the IC_50_ value of carvacrol was in the range of 53–151 µg/mL (25). 

Effect on ATP concentration**: **One of the antibacterial mechanisms is that *O. vulgare* EOs in combination with gamma radiation has an effect on periplasmic peptidoglycan composition and ATP concentration of *Listeria monocytogenes, Escherichia coli and Staphylococcus aureus*, which leads to cell wall damage (79-81).

*Effective preparation*

Decoction: Decoction is a method of extraction by boiling hard plant material such as roots, bark, seeds, and wood to primarily extract the mineral salts and bitter principles of plants. It was found that decoction did not possess any antibacterial effect against all isolates (82); because decoction consists of maximal levels of flavonoids and total phenolic compounds (rosmarinic acid) that gave higher antioxidant activity (20). 

Infusion: Infusion is the process of extracting chemical compounds from soft ingredients like leaves, flowers and citrus. Plant materials are suspended in hot water and closed the head of the extraction dish; the short brewing time helps to retain the vitamins and volatile ingredients while drinking. Some studies showed that the infusion was more effective against *Brevibacillus laterosporus* and *Bacillus polymyxa* (17.5-17.0 mm respectively) than the EO of *O.vulgare* against *Staphylococcus saprophyticus* and *Bacillus circulans* (16.8-14.5 mm respectively), while decoction has no antibacterial activity (83). Chaudhry *et al. *(2007) found that the antibacterial activity of *O.vulgare* infusion was similar to the EO (*Citrobacter* spp. 24 mm) and exhibited significant inhibitory activity against *Klebsiella pneumoniae, Klebsiella ozaenae *and *Enterobacter aerogenes* (20.1, 19.5and 18 mm) (82). 

Extraction: *O.vulgare* extracts (cyclohexane, dichloromethane and methanol extracts) have a moderate antimicrobial activity (MIC 62.5-125 µg/mL) against *S. aureus, Staphylococcus epidermidis, M. luteus, Bacillus subtilis, Enterococcus feacalis, K. pneumoniae, P. aeruginosa *and* Salmonella abony*. A. Cyclohexane extract has no activity against *Helicobacter pylori*, while dichloromethane and methanol extracts were active (MIC 250-500 µg/mL) (84). Methanol extract consists of the bioactive alkaloid (1.5%) and flavonoid (2.5%), was significantly active against multidrug-resistance (MDR) strains (*S. aureus, P. aeruginosa, E. coli*) isolated from the sore throat of patients (MIC 3.91 to 15.63 µg/mL) (85). In another study, significant antimicrobial activity against ulcer-associated *H. pylori* (ZI 10-15 mm) was found dependent on the high percentage of phenolic compounds and rosmarinic acid (100 μg per disk) in ethanol extract of* O. vulgare *(86). Licˇina *et al. *(2013) found that the potential inhibitory effect was showed by water extract against 19 strains bacteria (MIC <0.16–5 mg/mL). *S. aureus* and *Bacillus spp.* strains were sensitive to all extracts (water, acetone, ethanol, diethyl ether and ethyl acetate) with MIC values of 0.16–0.6 mg/mL (7). Akrayi *et al. *reported the low antibacterial activity of water extract (MIC < 12% v/v) against *P. aeruginosa,* *K. pneumonia, E. coli, *and* Proteus mirabilis *(87). In another study, inhibitory effects of methanolic extract of *O. vulgare* were evaluated toward ten bacterial and one *C. albicans* strains. Results showed that extract was active against *Porphyromonas gingivalis, Parvimonas micra* (MIC 0.3 mg/mL) and had almost no effect on *E. coli *and *C. albicans* (MIC 10 mg/mL) and reduced biofilm generation of *S. mutans* at 5.00 mg/mL (88).

Martins *et al. *using hydroalcoholic extract, decoction and infusion of *O. vulgare*, reported similar inhibitory effects against almost all the tested bacteria, while the hydroalcoholic extract showed relatively higher antibacterial activity against *E. coli* and *Proteus vulgaris* (20). The concentration used by Martins *et al. *(20 mg/mL) was noticeably less than the tested by those authors (200 and 100 mg/mL). It should be highlighted that EO contains antimicrobial substances (carvacrol and thymol) more than its methanol, ethanol, water and hexane extracts (89). 

*In-vivo antibacterial studies*

In an animal mouse model (n = 5 BALB/c mice, 2 MIC against *P. acnes* for 3 days, 2% erythromycin as positive control), a nanoemulsion with *O. vulgare EO* demonstrated to be effective on acne compared to the reference antibiotic (90). Antibacterial activity against *H. pylori* potential of a mixture of *Satureja hortensis* and *O. vulgare* ssp*.hirtum *EOs (2:1) was investigated *in-vivo *(n = 12 Balb/c mice, 2 Mix, for 5 days). By oral administration of this mixture, 70% of the animal group had been treated without any adverse effect or immune response that make this combination a safe and effective antibacterial treatment against *H. pylori* (91). 

*Antibacterial clinical trial*

The clinical trial (92) evaluated wound healing properties of *O. vulgare* extract ointment (3%, 40 patients undergone surgical excision) in comparison to the control group. The study proved that the ointment reduced bacterial contamination (*S. aureus, *22%) and infection on post-surgical wounds. 

*Antifungal effects*

*Mechanisms of action*

Effect on fungal cell wall: One of the mechanisms of antifungal activity is related to an attack on the cell wall and retraction of the mycelium cytoplasm and finally resulting in the death of hyphae. The EO of *O. vulgare* ssp. *virens* showed antifungal activity against human fungal pathogens (*Candida, Cryptococcus, Dermatophyte and Aspergillus strains*) with MIC values from 0.16 to 2.5 μL/mL. The result indicated that EOs lead to cell membrane disturbance, resulting in cell death. Antifungal potencies appeared to be enhanced in high carvacrol percentage, and the inhibition of filamentation correlated more with γ-terpinene content (40).

Fungal enzyme inhibition: Brondani *et al. *(93) demonstrated that *O. vulgare *EO at 1%, 5% and 10% (in DMSO) demonstrated significant reductions in phospholipase enzyme generation by *Candida albicans *(15 strains isolated from prosthetic stomatitis patients). Moreover, the mode of antifungal activity could be related to the EO components intervention in enzymatic reactions of cell wall synthesis affecting the morphogenesis and growth of fungal (94). 

*Anti-Candida activity*

The efficacy of *O. vulgare* on *Candida* species was proved by Stiles *et al. *(95) for *O. vulgare *and nystatin against* Candida* isolates obtained from human stools (40–45 and 22–25 mm, respectively). Cleff *et al. *(96) studied the effect of *O. vulgare* against reference strains of *Candida* and found that all were susceptible to the EO (MIC 1.2-5 μL/mL). Rosato *et al. *(97) (11.9 mm for *O. vulgare* and 17.8 mm for Origanum vulgare+Nystatin) and Souza *et al. *(94) (MIC 80 μL/mL for EO, 50 μL/mL for ketoconazole) observed that the *O. vulgare* EO inhibited all the *Candida* species in their study. In Bhat *et al. *study, hydrodistillation was a suitable extraction method and MIC was 0.024% which was much lesser than for fluconazole (0.25%) and the active functional group was carvacrol usually found in antifungal herbs (98). Another study showed that the antifungal effect of nystatin on *Candida albicans* was more than that of aqueous and alcoholic extracts of *O. vulgare *(99)*. In-vitro *investigation showed strong antifungal (*C. albicans* strains MIC 36-57 µg/mL) activity than antibacterial activity (MIC 64-120 µg/mL), of* O. vulgare *spp. *glandulosum* EO (100).

*Effect against other fungi*

*In-vitro *study of *O. vulgare* EO and its major constituents revealed the highest antifungal activity for γ-terpinene with MIC ranging from 62 to 500 µg/ mL against *Sporothrix schenckii*, and 125 to 250 µg/mL against *Sporothrix brasiliensis* (101). In contrast, the results of another study showed significant bacterial activity but a weak antifungal effect of the O*. vulgare* EO (7). 

*Antiviral activity*

The *O. vulgare* EO evaluated in the Meneses *et al. *study showed antiviral activities (CC_50 _< 100 µg/mL and MIC 3.7 µg/mL) against yellow fever virus via direct virus inactivation (102). Other reports include *O. vulgare *EO, toward murine norovirus and feline calicivirus with inactivation rates of 1.62 and 3.75 log, respectively (103). Treatment of equine arteritis virus resulted in a significant reduction in viral particle production (6.08 to 1.75^ log^ in the presence of 100 µL ethanolic extract of *O. vulgare*). Among the main compounds evaluated, quercetin was the most prominent as incubation reduces virus titer (10^0.6 ^TCID50/100 µL) (104). Other reports of the antimicrobial activity of *O. vulgare* have been summarized in Table S4 (in supplementary file). 

Safety and side effects

The *O. vulgare* EO and its main constituents, carvacrol and thymol, have been classified Generally Recognized as Safe (GRAS) for human usage by the US Food and Drug Administration (FDA) and European Parliament has approved culinary consumption (EP) and Council (105, 106). However, must keep in mind that EO of *O. vulgare* can be considered safe when used correctly and with precaution because of the toxic effects of carvacrol and thymol concentrated in the essential oil (20). There are a few reports on the adverse effects of *O. vulgare* essential oil (107). 

## Conclusion

The result of this survey will be helpful in the utilization of *O. vulgare* as a source of useful bioactive compounds. Large numbers of *O. vulgare* species are phytochemically investigated and results showed that their essential oil and extracts possess variable constituents and concentration that can be dependent on diverse factors such as species, soil conditions, climatic, harvest season, geographical location, growth conditions and extraction technique which emphasize the need to standardize quality control studies in the production of *O. vulgare* preparations. The EO of *O. vulgare *is the most investigated, and fascinating results have been reported, especially concerning its antimicrobial activity attributed to two main categories of phytochemicals: 1) Volatile compounds: EO comprises a large number of phytochemicals specially carvacrol and thymol. 2) Non-volatile phenolic compounds: Rosmarinic acid as phenolic acids is abundant in *O. vulgare *extracts. In addition, Flavones are the main flavonoids and luteolin is the most common one followed by apigenin.

Different studies demonstrated the remarkable antimicrobial effect of *O. vulgare *against a range of bacteria and fungi, especially MRSA*, E*. *coli *and *C.albicans*. Carvacrol and thymol showed a strong antimicrobial effect, especially against resistant microorganisms. For as much as thymol and carvacrol are volatile compounds, so infusion or tea products of *O.vulgare* have more amounts of these volatile ingredients and more effective than decoction and different extracts of *O.vulgare. *Furthermore, essential oil and different extracts are typically more effective than pure compounds because of the synergistic effect and mechanism of action involving different targets rather than a single mechanism. Consequently, further studies are required to identify various mechanisms of action and the effective dosage of EOs for clinical trials.

Finally, we can summarize our results as follows: *O. vulgare* appears as a particularly interesting platform for development into possible consumption in modern antibacterial products from ethnomedical traditions. The most investigated subspecies of *O. vulgare *is *Hirtum* and traditional uses reported for all subspecies have been confirmed by *in-vitro *and *in-vivo *antimicrobial studies, even if further studies are required for clinical trials. The major limitation in this research is the lack of well-designed, placebo-controlled, randomized clinical trials that can improve our current knowledge on the efficacy of *O. vulgare *ssp. in humans. The *O. vulgare* EO and its main constituents have been classified Generally Recognized as Safe (GRAS) for human usage by the FDA and traditional preparations and uses that do not show relevant toxicological properties.

 To expand and promote research on *O. vulgare* and its subspecies, the following approaches could be considered of value: standardize quality control studies in the production of various *O. vulgare* preparations; identify different mechanisms of action and the effective dosage of EOs for clinical trials; explain the biosynthetic pathways, the pharmacokinetics and pharmacodynamics properties (absorption, distribution, metabolism and excretion) and the toxicities (chronic and acute toxicity studies) of compounds present in *O. vulgare *and its subspecies; further studies for the use of *O. vulgare *various extracts, fractions or pure compounds as effective antimicrobial agents; design new studies concerning the traditional uses and scientific researches for the development of new perspectives for design a of new drugs. 

## Conflict of interest

All authors involved have no commercial association or other arrangements that might pose or imply a conflict of interest in connection with the submitted article.

## Supplementary Materials


