# Supplementary material for: A Review of the Phytochemistry and Antimicrobial Properties of Origanum vulgare L. and Subspecies
Source: Iran J Pharm Res. 2021 Spring;20(2):268–85. doi: 10.22037/ijpr.2020.113874.14539 (PMC8457725; doi:10.22037/ijpr.2020.113874.14539)
Supplement: Supplementary file 1 [file ijpr-20-268-s001.pdf]

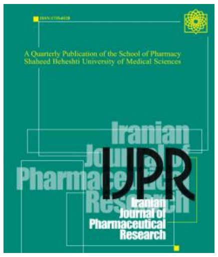

Supplementary Materials for

Synthesis, Kinetics, Binding Conformations and Structure-activity Relationship of Potent Tyrosinase Inhibitors: Aralkylated 2-Aminothiazole-Ethyltriazole Hybrids

Saba Soltani, Abolfazl Shakeri, Mehrdad Iranshahi and Motahareh Boozari*

*To whom correspondence should be addressed. E-mail: bouzarim@mums.ac.ir

Volume 20, Issue 2 (Spring 2021)

This PDF file includes:

Figures S1-S2

Tables S1-S4

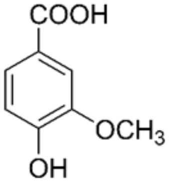

Vanillic acid (1)

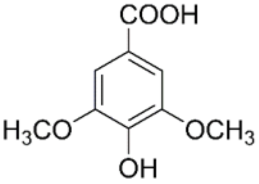

Syringic acid (2)

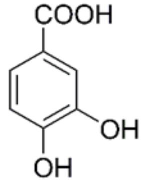

Protocatechuic acid (3)

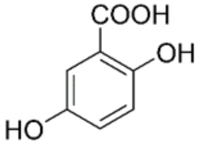

Gentisic acid (4)

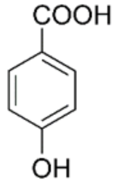

p-hydroxybenzoic acid (5)

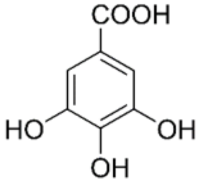

Gallic acid (6)

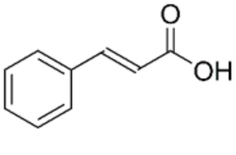

Cinnamic acid (7)

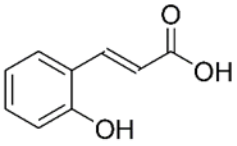

P-Coumaric acid (8)

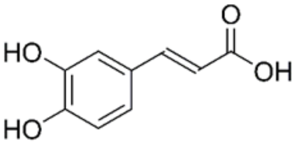

Caffeic acid (9)

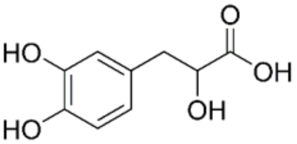

Danshensu (10)

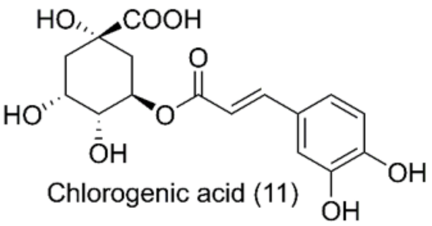

Chlorogenic acid (11)

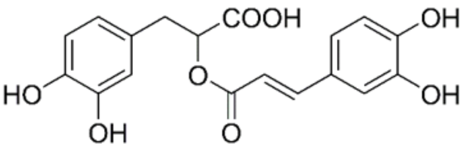

Rosmarinic acid (12)

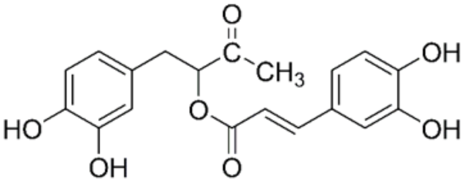

rosmarinic acid methyl ester (13)

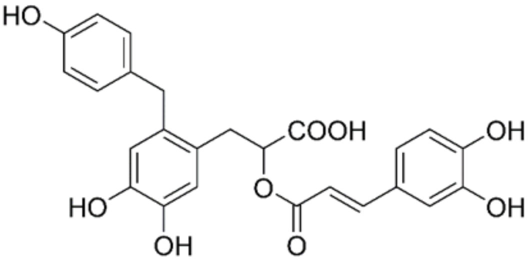

2-caffeoyloxy-3-[2-(4-hydroxybenzyl)-4,5-dihydroxy]phenylpropionic acid (14)

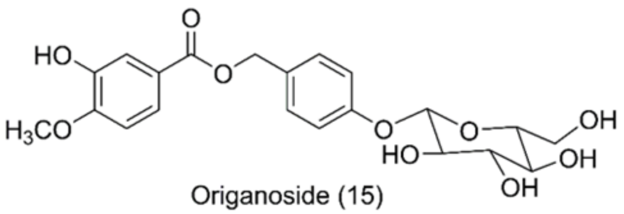

Origanoside (15)

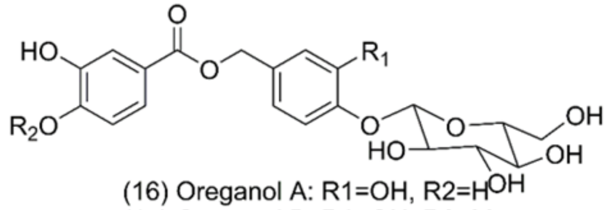

(16) Oreganol A: R1=OH, R2=H
(17) Oreganol B: R1=OH, R2=Me

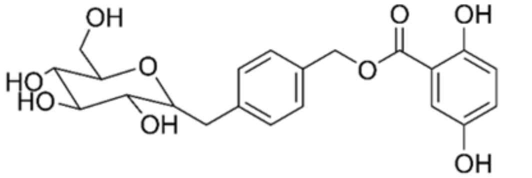

4-[[(2',5'-dihydroxybenzoyl) oxy] methyl] phenyl-O-b-D-glucopyranoside (18)

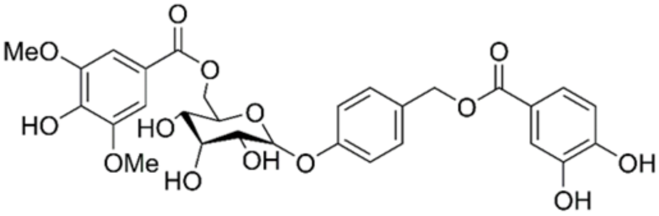

4-[[(3',4'-dihydroxybenzoyl) oxy]methyl]phenyl-O-b-D-[6-O-(3'',5'' O-dimethoxyl-4''-hydroxybenzoyl)] Glucopyranoside (19)

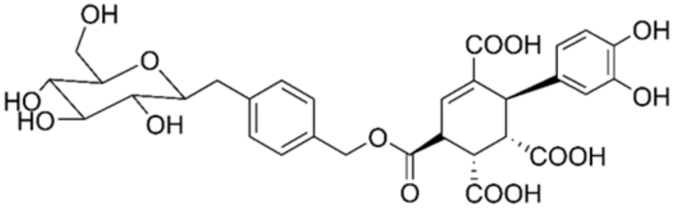

Origanine A (20)

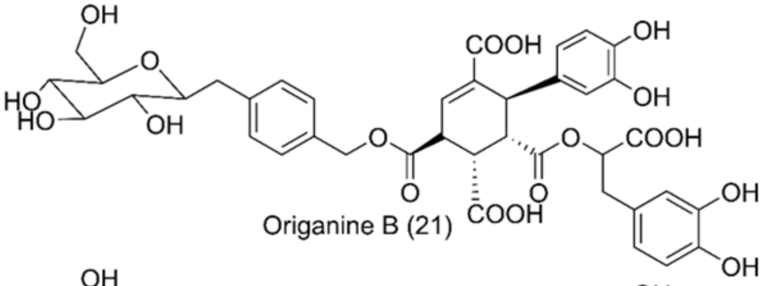

Origanine B (21)

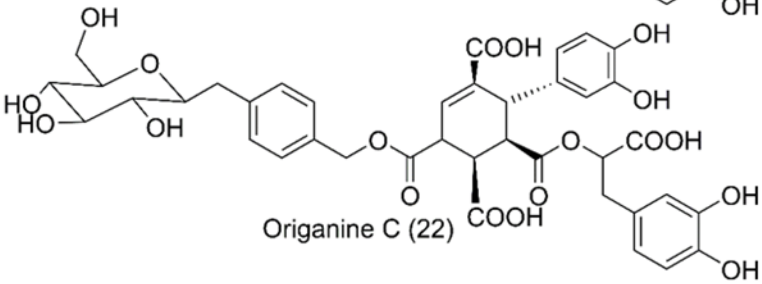

Origanine C (22)

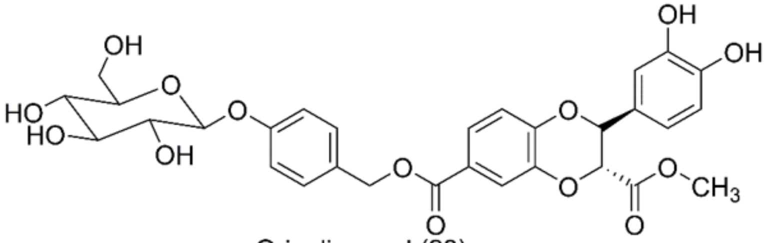

Origalignanol (23)

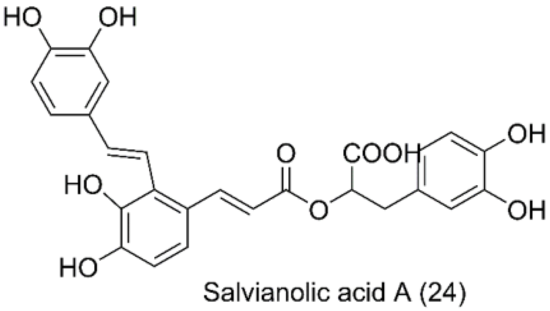

Salvianolic acid A (24)

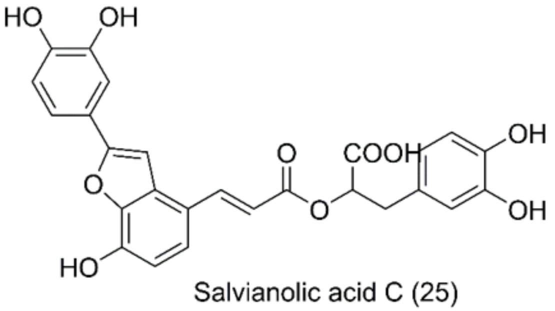

Salvianolic acid C (25)

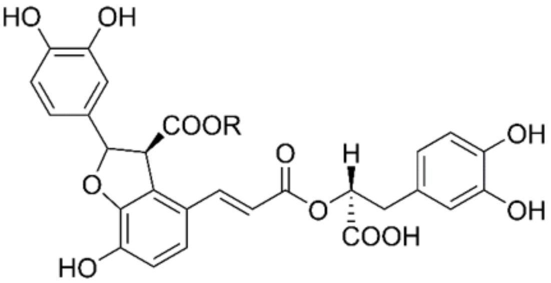

(26) 10-epi-lithospermic acid: R=H
(27) epi-lithospermic acid B : R=Caffeic acid
(28) lithospermic acid B: diastereomer of 27

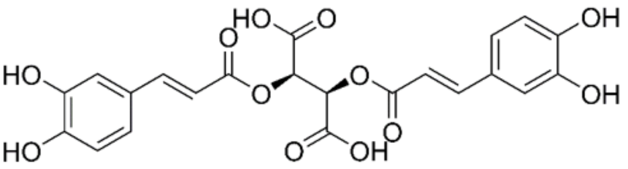

chicoric acid (29)

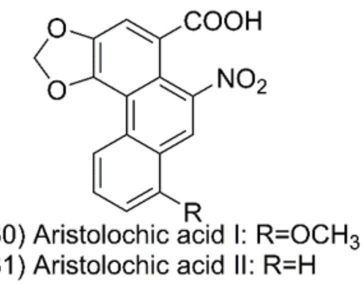

(30) Aristolochic acid I: R=OCH_3
(31) Aristolochic acid II: R=H

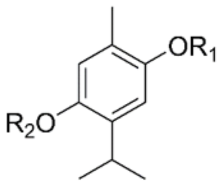

(32) Thymoquinol 2-O-b-glucopyranoside: R1= Glu R2= H
(33) Thymoquinol 5-O-b-glucopyranoside: R1= H R2= Glu
(34) Thymoquinol 2,5-O-b-diglucopyranoside R1= Glu R2= Glu

Figure S1. Chemical structures of main non-volatile phenolic compounds of *O. vulgare*.

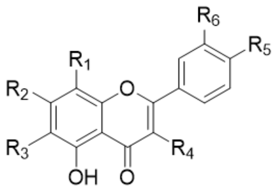

	R1	R2	R3	R4	R5	R5
(35) Apigenin	H	OH	H	H	OH	H
(36) Luteolin	H	OH	H	H	OH	OH
(37) Chrysoeriol	H	OH	H	H	OH	OCH_3
(38) Diosmetin	H	OH	H	H	OH	OCH_3
(39) Quercetin	H	OH	H	OH	OH	OH
(40) Cosmoside	H	O-Glu	H	H	H	OH
(41) Vicenin-2	C-Glu	OH	C-Glu	H	H	OH
(42) Vitexin	C-Glu	OH	H	H	OH	H
(43) Isoquercetin	H	OH	H	O-Glu	OH	OH
(44) Kaempferol	H	OH	H	OH	OH	H
(45) Cynaroside	H	O-Glu	H	H	OH	OH
(46) Rutin	H	OH	H	O-Rut	OH	OH
(47) Astragalin	H	OH	H	O-Glu	OH	H
(48) Hyperozide	H	OH	H	O-Gal	OH	OH
(49) 4'-methoxy Quercetin	H	OH	H	OH	OCH_3	OH
(50) Luteolin 7-O-glucuronide	H	O-glucuronide	H	H	OH	OH
(51) Apigenin-7-O-glucuronide	H	O-glucuronide	H	H	OH	H
(52) Luteolin-7-O-rutinoside	H	O-Rut	H	H	OH	OH
(53) Apigenin-7-O-rutinoside	H	O-Rut	H	H	OH	H

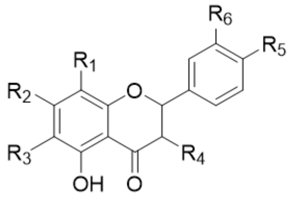

	R1	R2	R3	R4	R5	R5
(54) Eriodictyol	H	OH	H	H	OH	OH
(55) Taxifolin	H	OH	H	OH	OH	OH
(56) Naringenin	H	OH	H	H	OH	H

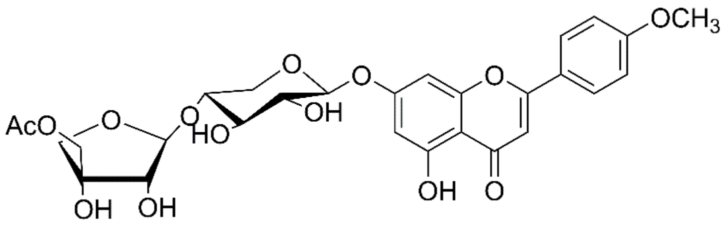

(57) Acacetin 7-O-[4¢¢¢-O-acetyl-b-D-apiofuransyl-(1®3)]-b-D-xylopyranoside

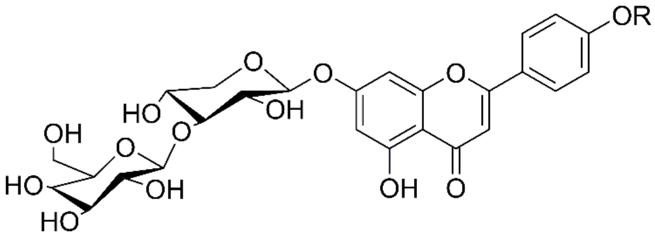

(58) Acacetin 7-O-[6¢¢¢-O-acetyl-b-D-galactopyranosyl-(1®3)]-b-D-xylopyranoside: R= CH3
(59) Apigenin 7-O-[6¢¢¢-O-acetyl-b-D-galactopyranosyl-(1®3)]-b-D-xylopyranoside: R= H

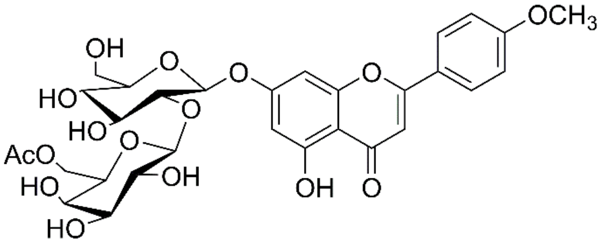

(60)Acacetin 7-O-[6¢¢¢-O-acetyl-b-D-galactopyranosyl-(1®2)]-b-D-xylopyranoside

Figure S2. Chemical structures of main flavonoids constituents of *O. vulgare.*

Table S1. Summary of traditional uses of *O.vulgare* ssp.

Scientific name, local name	Origin	Part used	Traditional uses	Method of utilization	Reference
O. Vulgare, vranilova trava	South-western Serbia, zlatibor district	Leaf	Urinary tract infections	Infusion	(108)
O.vulgare, vranilovka, crnovrška, origano	Suva planina mountain region (south-eastern serbia)	Arial part	Pulmonary and stomach diseases, colds, kidney and urinary infections, improving appetite, sedative, tonic	Tea	(109)
O. Vulgare, vranilova trava	Eastern Serbia	Aerial part	Immune system strengthening, digestive, hypolipemic	Infusion	(110)
O. Vulgare, çaj bieshke planinski čaj	Western Balkans and south-western Serbia	Flowering aerial parts, dried	Stomachache, digestive, vaginitis, panacea	Tea	(111)
O. Vulgare, za'atar	South-eastern morocco	Leaf, stem	Diabetes, hypertension	Infusion	(112)
O. Vulgare, sahtar, essahtar	Mokrisset region (nw of morocco)	Leaves and inflorescence	Hypertension, diarrhoea	Additive for figs, olives and coffee, pastoral	(113)
O. Vulgare	South-east region of morocco (tafilalet)	Leaves	Hypertension and cardiac diseases	Infusion	(114)
O. Vulgare	Northern Albania	-	Digestive, kidney	Infusion	European project rubia (115)
O. Vulgare, tcherven rigan	Bulgaria	Aerial part	Antitussive, stimulates gastric secretion, choleretic, cholagogue	Infusion	(116)
O. Vulgare, oregano	Italy	Aerial part	Aperitive, digestive. Mild antiseptic, spasmolytic, expectorant, in case of inflamed lungs	Infusion, tincture	(116)
O. Vulgare	Russia and central Asia	Aerial parts	Pyoderma, skin wounds, skin irritations, allergic rashes and dermatitis	Galenical and essential oil	(117)
O.vulgare, kakelik-oti	Turkmen sahra, north of Iran	Aerial parts	Kidney stone, colitis, food digestion	Flavoring with yugart	(118)
O.vulgare, te de roig, t ´ e roig, t ´ e rojo	Castellon, Spain	-	Depurative, antirrheumatic, anti-inflammatory, stomachic	Tea	(119)
Ssp. *Glandulosum*, zâtar	M'sila (north Algeria)	Aerial part	Antihypertensive, digestive disorders antidiabetic	Decoction	(120)
Ssp. *Glandulosum*, zâtar	Setif region (north-eastern Algeria)	Aerial part	Whooping cough, cough, fever and bronchitis Externally applied to relieve rheumatic pains	Decoction+ powder	(121)
Ssp. *Gracile*, Kekik	Turkey Nakhchivan (Azerbaijan)	Flowering tops	Stomachic, diuretic, digestive, mouth and teeth disease, dermal respiratory, neurological, Vulnerary, hypertension, epilepsy, headache, asthma, Cold	Infusion, Decoction	(122-124)
Ssp. *Hirtum*, rigani	Thessaloniki (Greece)	Aerial parts	Diabetes constipation, bloating, diarrhoea, dyspepsia, spasmolytic, cholesterol, neuralgia, toothache, asthma, Common cold, rheumatisms, antiseptic, Dysmenorrhoea, Antipyretic, stimulant	Chewing, infusion, decoction, external application (footbath, washings), seasoning	(125)

Ssp. *Hirtum*, arigane	Southern Italy	Flowering tops	Toothache	Smoked, fumes inhaled	(21)
Ssp. Virens	Brazil	Leaves	Analgesic and expectorant	Infusion	(126)
Ssp. Virens ourego, origano	Northwest Spain	Aerial part	Anticatarrhal; to reduce pain after childbirth	Infusion	(127)
Ssp. *Virens*	Southern Spain	Flowery plant	Cold, cough, odontalgy, digestive disorder, food poisoning	Infusion or decoction	(128)
Ssp. *Viridulum*	Turkey	Flowering tops	Stomachic, dispepsia	Decoction	(122, 124)
Ssp. *Viridulum*, Areana	Southern Italy	Leaf	Sedative, against insomnia	Infusion	(129)
Ssp. *Vulgare*, tsai dramas, pontiako tsai, kokkino tsai	Greece	Aerial parts	Bloating, calmative, Common cold, Appetiser, stimulant	Infusion	(125)
Ssp. *Vulgare*, kekikotu, keklikotu, kekli/ ssp. *Hirtum*, yer kekigi, kekikotu, keklikotu, keklik	Turkey	Aerial parts	Cold, flu, Abdominal pain, kidney stones and stomach diseases	Decoction	(130)

Table S1. Chemical diversity in the essential oil composition of *Origanum vulgare* ssp. reported from different regions.

Essential oil	location	Oil yield (w/w%)	Main components	reference
ssp. glandulosum	Eastern Algeria	2.2%	Thymol (70.59%), t-butyl benzene (5.76%), carvacrol (5.44%)	(31)
ssp. glandulosum	North of Tunisia	Nefza : 4.3-5.8%	Thymol (31.8-46.1%), γ-terpinene (24-27.1%), *p*-cymene (11.5-35.7%)	(30)
		Krib: 2-2.7%	*p*-cymene (29.1%–46.3%), thymol (18.4%–31.5%) ,γ -terpinene (16.1%–23.5%)	
ssp. glandulosum	Tunisia	Early vegetative stage: 1.7%.	carvacrol (61.08%), *p*-cymene (9.87%), γ-terpinen (6.34%), borneol (2.38%)	(29)
		Late vegetative stage: 2%	carvacrol (67.93%), *p*-cymene (5.40%), γ- terpinen (4.25%)	
		Flowering stage: 0.6%	carvacrol (83.37%), γ-terpinen (4.13%), *p*-cymene (3.02%)	
ssp. gracile	Iran	Leaves and flowers (1.44% and 2.44%)	Carvacrol (46.5% and 60.6%), γ-terpinene (13.91% and 16.64%), ρ-cymene (13.54% and 7.21%)	(32)
ssp. gracile	France	0.7%	sabinene 26%, germacrene D 14%, β-caryophyllene 7%	(34)
ssp. gracile	Turkey	0.04%	β-caryophyllene (17.54%), germacrene D(12.57%)	(33)
ssp. hirtum	Greece	1.0%	Carvacrol (60.82%), thymol (3.53%), p-cymene (6.58%),	(131)
ssp. hirtum	southern Italy	2.7%	Thymol and Carvacrol (84.7%), E-caryophyllene (3.2%)	(132)
ssp. hirtum	Moldova	2.409- 5.422%	carvacrol (67.67-85.85%), p-cymene (3.64-9.33%), γ-terpinene (8.22%)	(133)
ssp. hirtum	Turkey	7.31%	Linalool (96.31%), β-caryophyllene (1.27%)	(37)
ssp. hirtum	Turkey	3-6.1%	carvacrol (85.4–5.3%), thymol (68.0–0.3%), *p*-cymene (31.6–2.8%	(134)
ssp. hirtum	Bulgaria	2.3- 7.4%	Carvacrol (56.3-78.8%), γ-terpinene (4.3-18.9%), p-cymene (4.1-17.8%)	(135)
ssp. virens	Spain	Leaves- flowers (8.3 -15.1 μg/g)	Linalool (26.2,38.2%), cis-Ocimene (15.3, 4.7%), Sabinene (7.3,3.5%)	(136)
ssp. virens	France	0.8%	germacrene D 26%, γ-Terpinene 13%, thymol 10%, Sabinene 6.5%	(34)
ssp. virens	Iran	0.2-0.5%	(Z)-α-bisabolene 39.17%, β-bisabolene 4.24%, sabinene 11.52%, (Z)-β-ocimene 2.39%	(4)
ssp. virens	Portugal	4.5%	Carvacrol (68.1%), γ-Terpinene (9.9%), *p*-cymene (4.5%)	(39)
ssp. virens	Central Portugal	1.2%	α-terpineol (65.1%), γ-terpinene (34.2%), carvacrol (34.2%)	(40)
ssp. virens	Mediterranean populations	2.8%	Linalool (76.8%), Carvacrol (3.7%), bicyclogermacrene (2.9%)	(41)
ssp. viridulum	Iran	9.5 -10.6%	Thymol (28-32%), 4-terpineol (10-12%), α terpinene (7.5-10%)	(137)
ssp. viridulum	Iran	0.4%	Thymol (29.9%), γ-terpinene (13.0%), β-pinene (11.3%)	(46)
ssp. viridulum	Turkey	-	Caryophyllene oxide (25.01%), linalool (8.32%), 1,8-cineol (7.98%)	(44)
ssp. viridulum	Greece	8.8%-5.4% (flowers-leaves)	Thymol (61%-69%), carvacrol (11.3%-10.4%), (Z)-β-ocimene and γ-terpinene (10.3% - 4.7%)	(138)
ssp. vulgare	Turkey	5.09%	Thymol (58.31%), carvacrol (16.11%), *p*-Cymene (13.45%)	(37)

ssp. vulgare	Mediterranean oregano populations	0.3%	Sabinene (16.3%), (13.3%), β-caryophyllene (10.7%)	(41)
ssp. vulgare	Argentina	-	O-cymene (14.25%), terpinen-4-ol (12.48%), E-β-terpineol (10.4%), thymol (10.13%)	(39)
ssp. vulgare	India	0.2% -2.1%	thymol (13.7–85.9%), carvacrol (1.5–63.0%), β-caryophyllene (0.2–13.8%)	(139)
ssp. vulgare	Bulgaria	0.06%	Spathulenol (20.7), *β*-caryophyllene (9.9) and caryophyllene oxide (5.7)	(140)
ssp. vulgare	Iran	0.5%	thymol (37.13%), γ-terpinene (9.67%), carvacrol (9.57%)	(141)
ssp. vulgare	Moldova	0.108 - 0.249%	GermacreneD(17.01%), β-caryophyllene (13.05%), carvacrol (11.65%)	(133)
ssp. vulgare	374 individual plants of Austria	0.1 - 1.8%	sabinene (up to 48.4%), cis-sabinene hydrate (up to 57.8%), p-cymene (up to 49.7%)	(24)
ssp. vulgare	Italy	lowest-altitude (1.33 and 1.05%)	(E)-β-ocimene (10.4 – 17.5%), (Z)-β-ocimene (6.6 – 19.0%), germacrene D (11.1 – 20.7%)	(142)
		intermediate-altitude (1.18 and 0.96%)	(Z)-β-ocimene (6.6 – 19.7%), (E)-β-ocimene (9.5 – 10.4%), thymol (6.5 – 9.9%), germacrene D (7.9 – 9.7%),	
		highest altitude (0.96 – 0.82%)	carvacrol (31.5 – 40.0%), γ-terpinene (24.4 – 38.5%), (E)-β-ocimene (3.2 – 5.7%)	
ssp. vulgare	Lithuania	Inflorescences- leaves (0.1–0.4%)	β-ocimene (13.3-23.3%), sabinene (13.3%-8.3%), germacrene D (13%, 14.3%)	(143)

Table S2. Flavonoids and phenolic acids constituents of *Origanum vulgare* ssp. reported from different regions.

Oregano Species	Origin	Flavonoids and Phenolic Acids Constituents*	Reference
ssp. *hirtum*	Greece	12, 25, 28, 54, 56	(144)
ssp. *hirtum*	Greece	9, 12, 26, 27, 28, 32, 33, 34, 35, 36, 37, 38, 39, 40, 41, 54	(145)
ssp. *hirtum*	Greece	11, 12, 35,54, 55, 56	(146)
ssp. *hirtum*	Massachusetts	12, 14, 27, 28, 41, 50	(147)
ssp.*virens*	Argentina	3, 9, 10, 12, 15, 16, (35, 36glycosides)	(148)
ssp. *viridulum*	Turkey	6, 8, 9, 12, 29,39,40, 44, 56	(44)
ssp. *vulgare*	Romania	1, 2, 3, 5, 9, 11, 12, 35, 36, 50 , 56	(51)
ssp. *vulgare*	Romania	4, 8, 11, 12, 36, 39, 46, 48	(149)
O. vulgare	Turkey	1, 2, 3, 6,8,11, 12, 35,36, 39,40,43,45, 46,49,52,53	(150)
O. vulgare	Russia	30, 31	(151)
O. vulgare	Poland	9, 11	(152)
O. vulgare	China	15	(58)
O. vulgare	Japan	16, 17	(153)
O. vulgare	Massachusetts	3, 8, 9, 12,39	(86)
O. vulgare	Lithuania	9, 11, 12, 36, 39, 38, 42, 46, 47, 48, 54, 56	(50)
O. vulgare	China	2, 3, 5, 8, 9, 11, 12, 39	(52)
O. vulgare	India	16, 17	(61)
O. vulgare	Taiwan	23, 24, 25, 26, 36,45, 50, 51	(63)
O. vulgare	Poland	1, 3, 5, 9, 12, 35, 36, 39, 44, 45,46	(49)
O. vulgare	China	20, 21, 22	(62)
O. vulgare	China	1, 3, 9, 12, 39	(154)
O. vulgare	China	3, 4, 12, 15, 18,19, 57, 58, 59, 60	(59)

*The numbers of the flavonoids and phenolic acids constituents are those of Figure 2 (in main file).

Table S4. Antimicrobial activities of *O. vulgare*.

**Model organism**	**Extract type**	**Model used**	**Positive/Negative control**	**Results**	**Ref.**
S. epidermidis	EO of ssp. *hirtum*	Broth dilution method (DRD: 0.8 to 100 µg.mL^{-1} at 37 °C for 24 h)	Gentamycine/ Sterile medium	MIC: 25-100 µg/mL	(155)
S.maltophilia, C. luteola	EO of ssp. *hirtum*	Disc diffusion method (DRD: 20µl per disc at 30 °C for 48 h for yeast, at 37 °C for 24 h for bactria)	penicillin, ampicillin, amoxicillin+ clavulanic acid, imipenem, cefoperazone, methicillin, oxacillin, gentamicin and nystatin/NA	30 - 28 mm	(156)
Klebsiella oxytoca	EOs of ssp. *hirtum*	Micro dilution method (DRD: 512 to 0.125 µg.mL^{-1} at 37 °C for 16 h)	NA	MIC: 0.90 - 2.11 µg/mL	(157)
E. coli, L. anguillarum,Vibrio sp., V. splendidus, V. alginolyticu, S.cerevisiae	EOs of ssp. *hirtum*	Disc diffusion method (DRD: 2 µl per disc at 35 °C for 24 h)	O/129, Ampicillin, Miconazole / Vehicle	7.3-18.8 mm	(158)
Salmonella thyphimurium	EO of ssp. *gracile*	Micro dilution method (DRD: 62.5 to 1000 ppm at 37 °C for 24 h)	NA/ Sterile medium	125-250 ppm	(32)
Bacillus subtilis	EO of ssp. *glandulosum*	Micro dilution method (DRD: 250 to 3.90 µg .mL^{-1} at 37°C for 24 h)	Sterile medium plus inoculum / EO plus Sterile medium	125-250 µg/mL	(29)
S. epidermidis	EO of ssp. *virens*	Broth dilution method (DRD: 100 to 0.8µg.ml^{-1} at 37 °C for 24 h)	Chloramphenicol/ Sterile medium	3.12-25 µg/mL	(159)
Sarcina lutea,C. albicans	EOs of ssp. *vulgare* and ssp. *hirtum*	Micro dilution method (DRD: 0.5 to 512 µg.ml^{-1} at 37 ◦C for 18–24 h for bacteria and 30 ◦C for 24–36 h for *C. albicans*)	NA/Vehicle	*Ssp. vulgare S. lutea* (85.3 µg/mL), *ssp. hirtum C. albicans* (85.3 µg/mL)	(37)
40 microorganisms (24 bacteria and 15 fungi and yeast)	EO of ssp. *Vulgare*	Micro dilution method (DRD: 7.8-500 µg.ml^{-1} at 37 ºC for 724h)	Amphotericin B/ Vehicle	MIC :15.62 to 125 µg/mL	(89)
S. aureus, S. flexnery	*O.vulgare* EO	Optical density method (DRD: 0 – 4.6 mg.mL^{-1} at 35 ºC for 24h)	Chloramphenicol/NA	230 µg/mL	(160)
Acinetobacter baumannii, E. coli	*O.vulgare* EO	Broth macrodilution method (DRD: 4 to 0.125 (v/v) at 37 °C for 24 h)	NA	0.25-0.75% v/v	(161)
C. albicans, S. aureus, B, subtilis, E. coli, P. aeruginosa, A. niger	SFE extracts from *O. vulgare*	broth dilution method (DRD: 40 mg.mL^{-1} for 24 h for bacteria and 48 h for yeast)	Chloramphenicol and amphotericin B / Vehicle	MBC: 1480 ± 0.05 to 2850 ±0.15 µg/mL	(162)
S. aureus (MSS, MRS) strains	*O. vulgare* EO	Agar dilution method (DRD: 0.5% to 0.0035% (v/v) at 30 °C for 18-24 h)	NA/ Sterile medium	0.06–0.125%, v/v	(138)
L. innocua, L. monocytogenes	*O. vulgar* EO	microdilution method (DRD: 20 µL per disc at 30 °C for 24 and 48 h respectively)	NA/ Vehicle	MIC : 2200 µg/mL	(163)
B.subtilis	*O. vulgar* EO	Resazurin micro-titreplate method (DRD: 2.5 mg.mL^{-1}, w/v in 10% DMSO at 37 °C for 24 h)	Ciprofloxacin/ Vehicle	MIC : 70.0 µg/mL	(164)

S. aureus	*O. vulgar* EO/carvacrol/ methicillin	microdilution method	Methicillin/NA	625/312.5 /384 µg/mL	(165)
S. enteric , *L. monocytogenes*	*O. vulgar* EO	microdilution method (DRD: 0.3-5 µL/mL at 30 °C for 24h)	Sterile medium plus inoculum / Sterile medium	MIC : 0.6 -1.2 µL/mL	(166)
B. megaterium, E. coli	*O. vulgar* EO/carvacrol/Citral	disc diffusion method (DRD; 15 µL of 50 and 25% concentration per disc)	NA	45-20 mm	(167)
59 MDR strains of *S. maltophilia*, MRSA, *A. xylosoxidans*	*O. vulgar* EO	microdilution method (DRD: two-fold diluted from 2% to 0.007%, v/v at 37° C for 24.48,72 h)	NA/ Sterile medium	MIC: 0.015%, 0.25%, 0.5% v/v	(168)
S. aureus , *Shigella*	EOs of ssp. *hirtum*	Disc diffusion method (dilution factor 0.1-0.001)	NA	91.20-81.28AU/mL	(169)
C. albicans , MDR strains of *E. coli, C. famata, Enterococcus* spp.	*O. vulgar* EO	Broth microdilution method (DRD: 10% to 0.03% at 37°C for 24 h)	NA/ Sterile medium	0.09-1.18 mg/mL	(170)
S.thyphimurium, B. cereus, S. enteritidis, MRSA, *E. coli, S. aureus*	*O. vulgar* EO	Broth microdilution method (DRD: 2560 µg/mL to 1.25 µg/mL by two-fold dilution at 37 °C for 24 h)	Amikacin/ NA	MIC: 160- 640 µg\mL	(171)
P. acnes and *S. epidermidis*	*O. vulgar* EO	Broth microdilution method (DRD: serial two-fold dilutions from 0.00875–0.56% (v/v) at 37 °C for 48 h under anaerobic conditions and 25 °C for 24 h in aerobic conditions respectively)	Erythromycin and Clindamycin/ Vehicle	MIC: 672 and 1340 µg/mL, respectively Anti-biofilm effect against *S. epidermidis* (MBIC 1344 µg/mL)	(90)

DRD: Dose range tested and duration; NA: Not available.
